# Multi-Scenario Simulation Analysis of Grain Production and Demand in China during the Peak Population Period

**DOI:** 10.3390/foods11111566

**Published:** 2022-05-26

**Authors:** Xiang Wang, Wenli Qiang, Shuwen Niu, Anna Growe, Simin Yan, Nan Tian

**Affiliations:** 1 College of Earth and Environmental Sciences, Lanzhou University, Lanzhou 730000, China; xwang19@lzu.edu.cn (X.W.); shuwenn@lzu.edu.cn (S.N.); tiann20@lzu.edu.cn (N.T.); 2 Institute of Geography, Faculty of Chemistry and Earth Sciences, Heidelberg University, 69120 Heidelberg, Germany; anna.growe@uni-heidelberg.de (A.G.); simin.yan@stud.uni-heidelberg.de (S.Y.)

**Keywords:** demand scenarios, production projections, gap, population peak, China, food security

## Abstract

The transformation of dietary structure brought about by economic development in populous countries is expected to trigger an increase in grain demand, which will put enormous pressure on the grain supply in these nations and even globally. We simulated nine demand scenarios for 2020–2050 based on China’s dataset for 15 kinds of grains from 1961–2018. The results show that the maximum difference between the predicted grain demand is 323.8 Mt, equal to the total grain consumption of approximately 600 million Chinese people in one year. To capture which demand scenarios will be met when grain productivity gradually improves within reasonable ranges, we present three projections from the production side. In particular, Projection 1 (P1), which maintains productivity at the current level, only fulfills the projected demand for Scenarios 1-LL, 2-LM, 4-ML, and 7-HL and falls short of the maximum value (Scenario 9-HH) by 117 Mt, which requires an additional 250,000 ha of arable land resources to fill the gap. After raising the preset value of grain yield, the productivity of Projection 2 in turn satisfies the demand scenario 5-MM. When both set variables (grain yields and arable area) increase simultaneously, the output of Projection 3 increases by 15.3% over P1. However, it still lags behind the demand of 68 million tons in Scenario 9-HH, thus implying uncertainty in China’s vision of meeting the goal of 95% grain self-sufficiency. Rather than pursuing a single outcome, we discuss multiple possibilities for China’s future grain balance and emphasize the adjusting and compensating role of grain trade and storage in the whole system. Ultimately, this paper calls for a better understanding of the supply–demand gap therein and its future trends to support national grain security as well as global sustainable food policies.

## 1. Introduction

The past half-century has seen rapid economic growth, urbanization, population expansion, and dietary structure shifts, all of which have led to profound changes in global and regional grain demand [1,2,3]. The advancement of grain production, processing, and storage technologies and the increasingly powerful and globalized grain industry effectively support the global grain supply system [4,5]. However, profound changes in supply and demand have not only resulted in the continuous evolution of global and regional grain systems but have also become a major challenge facing the world [6,7]. By the mid-century, the global population will likely stabilize at approximately 9 billion people, and global grain production will have to increase by 50% by then to meet their needs [8,9,10,11]. This trend will be accompanied by a transition of the grain system from producer to consumer and the forces that control it. Currently, grain production is experiencing greater competition for land, water, and energy, and the need to curb its negative environmental impacts is becoming increasingly evident [12,13,14,15]. Beyond these, climate change, extreme natural disasters, and COVID-19 are also emerging as uncertainties that affect the grain supply system [16,17,18].

As the world’s most populous country, any change in China’s grain demand is likely to profoundly impact the global food system [19]. Arable land was only 8% of the world’s total, and water availability per person accounted for 1/4 of the global average, but China fed approximately 20% of the world’s population, which was both a remarkable achievement and a huge challenge [20,21]. The nutritional transition has led to a dramatic shift in the Chinese diet away from staples and increasingly toward livestock and dairy products, vegetables and fruit, and fats and oils [22]. However, the diversification of grain uses has caused a structural shortage of domestic grain supply, making China’s grain supply and its security issues of major interest to international markets and trading partners [23,24]. The main characteristic of China’s grain demand is that the consumption of food has been decreasing annually, but the increase in consumption of meat, egg and milk have caused the growth of feed. In light of the rapid economic development and population growth trends, Wang argued that the gap between grain supply and demand in China would further widen, thus putting new strains on the national grain supply [25]. In addition, recent rising wages significantly increased the cost of grain production, lowered agricultural competitiveness in the global market, and exacerbated food insecurity concerns in China [26]. With the gradual penetration and control of China’s grain production and trade system by international agricultural monopoly capital attempts, ensuring food security under the red line of resource constraints has become the main challenge to be addressed. When China’s population peaks, grain balance issues will put enormous pressure on its resources and environment and have a major impact on global agricultural markets and food security.

Over the past two decades, many existing studies and analyses have addressed the Chinese food security challenge ahead [27,28,29]. Some scholars have focused on growth drivers of China’s grain demand and prospects for grain production responses, projecting grain demand (cereal and soybean only) to be approximately 600–700 Mt in 2030 [30,31,32]. Some further projected Chinese grain demand to 2050, with assumptions on population growth and dietary shifts, estimating the volume of additional grain demand over the next 30 years [33,34,35]. According to UN estimates, China’s population is predicted to peak at 1.4–1.5 billion in 2025–2043, and the size of the population directly determines the total demand for grains [36,37]. Although previous studies analyzed the issue of “food safety” and “food security” in China from multiple perspectives, a comprehensive study to articulate the issue was absent. In this context, we review the historical grain production and demand trends in China, relate these changes to global trends, and simulate multiple scenarios using varying factors to discuss potential challenges to China’s future food security.

In this study, we attempt to develop a “past-present-future” research framework to match China’s future grain demand and production. Based on historical trends in grain supply and demand, we discuss grain supply from domestic production as well as grain storage, and analyze grain demand depending on grain use, dietary shifts, and grain waste. This study combines various possibilities of grain production and demand to examine the matches and uncertainties between these two sides. However, in the most important simulation section, we first estimate China’s grain demand multiplex scenarios at the peak population in combination with the per capita grain demand. Then, three different projections are used to predict domestic grain production, including two influencing variables, yield and the amount of arable land. Finally, we addressed forecasts and global implications by setting up different variable scenarios [38]. An analytic framework for food security in China is thus developed, and policy measures to regulate the supply–demand relationship are proposed. Specifically, this study focuses on the following issues:

1. Simulating China’s grain demand under different scenarios and analyzing their diversity and uncertainty.
2. Predicting potential projections for China’s grain production, with a stepwise overlay of factors affecting productivity.
3. Calculating the gaps between the grain demand scenarios as well as the production projections and proposing options to fulfill the balance.

## 2. Methods and Materials

Based on the economic theory of supply and demand, we designed this study to match its basic elements. The simulation of grain demand and supply in China during the population peak involves a large number of variables and a complex model structure, which requires a combination of several research methods.

(1) In the demand theory, the influencing factors, including income, grain price, preference, and population size, are adequately considered. In this regard, the price is inversely correlated with the quantity demanded. However, as grain is essential to meet the necessities of human beings, the elasticity of demand is small and the effect of price is not significant. Due to the diversity of grain uses, we employed a functional decomposition analysis to decompose grain demand into food (direct consumption), industrial use, feed, seeds, and losses. The direct consumption is extrapolated from the historical change trend. The indirect consumption was calculated based on the conversion rate estimation method, because feed and industry consume grain in a certain ratio. The per capita grain demand was estimated by combining the parameter debugging method.
(2) In the supply theory, the technological development, factor input and natural resource endowment are the main influences. Therefore, we chose the two main factors affecting production—yields and arable land area—to analyze the changes in the time series using panel data and a stepwise approach to increasing control variables.
(3) The demand side is less elastic than the supply side in this balance. Thus, the domestic production capacity, grain imports and stocks are the vital externalities to regulate supply. Comparing the demand and supply scenarios in an integrated manner, we addressed China’s potential risks and challenges in balancing grain supply and demand when facing the population peak.

**Figure 1:**
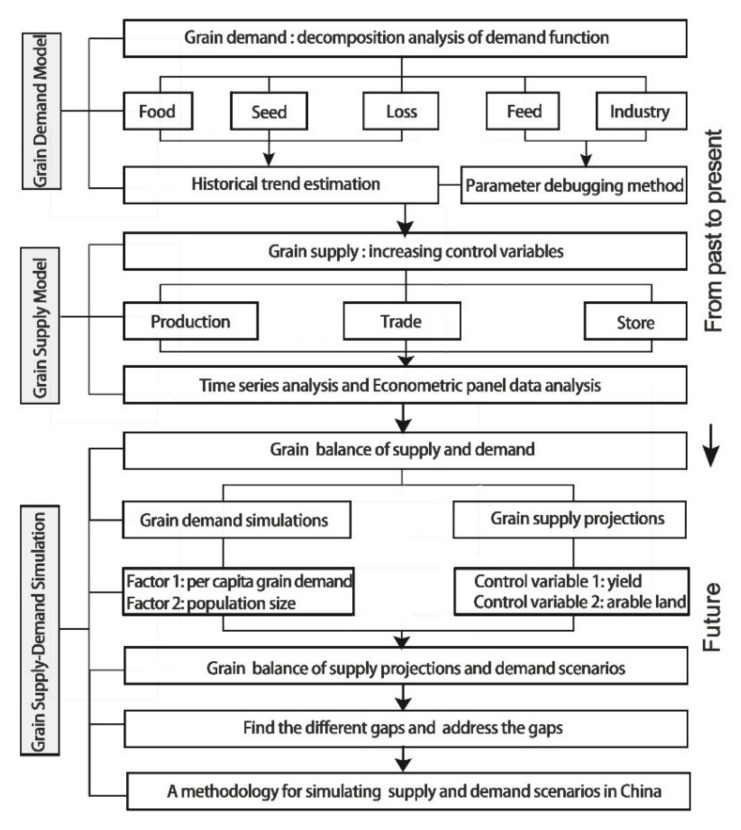
Research methodology.

In this way, the research contents are presented in three parts, including scenarios of grain demand, projections for grain supply, and simulations of the supply–demand balance. Each part has a corresponding analyzing path and specific methodologies and is jointly formed into a complete research framework, as detailed in Figure 1.

### 2.1. Grain Demand Model

Clarifying the structure of grain consumption is a prerequisite for forecasting grain demand. Based on the classification standards of Food and Agriculture Organization of the United Nations (FAO), grain demand can be divided into food, feed, industrial use, seed and losses [39]. The total grain demand (*D*) is calculated as follows:

(1) $$D=D_{1}+D_{2}+D_{3}+D_{4}+D_{5}$$

where *D*_1_, *D*_2_, *D*_3_, *D*_4_, and *D*_5_ indicate grains for food, feed, industrial use, seed and losses, respectively.

#### 2.1.1. Food Use

Food use is the portion of grain demand used for direct consumption. It accounts for 61% (using the multi-year average) of total grain demand. Taking into account the differences in ration consumption between urban and rural residents as well as the noticeable trend of eating out in China, we employed the following calculation:

(2) $$D_{1}=\overset{2}{\underset{i=1}{\sum }}P_{i}E_{i}+\overset{2}{\underset{i=1}{\sum }}P_{i}F_{i}$$

where *D*_1_ represents food use, *P*_i_ refers to urban and rural populations, *E*_i_ denotes the per capita grain demand of food use, and F_i_ is the frequency of urban and rural residents eating out.

#### 2.1.2. Feed Use

Feed grains are the portion of grain demand used to produce meat, eggs, milk, and aquatic products and represented 24% of the total demand. Owing to the improving residential living standards and the shifting dietary structure, the demand for feeding grains has altered as well, and it was calculated as follows:

(3) $$D_{2}=\overset{4}{\underset{i=1}{\sum }}C_{i}T_{i}$$

where *D*_2_ represents the demand for feed grains and *C_i_* represents the consumption of meat, eggs, milk and aquatic products. *T_i_* is the coefficient of grain consumption per unit of these products, which we converted according to the current Chinese ratios for meat (1:2.7), eggs (1:1.7), milk (1:0.4) and aquatic products (1:1). Since the production of livestock products indirectly consumes grains, the conversion of livestock products into indirect demand for grains is calculated.

#### 2.1.3. Industrial Use

Industrial grains mainly include grains demand for brewing, starch, soybean crushing and biofuel ethanol, which has strongly occupied 5% of the total demand in recent years. Along with the continuous development of China’s processing industry, the demand for industrial grains has become the third largest type of grain demand in China, and it was calculated as follows:

(4) $$D_{3}=\overset{5}{\underset{i=1}{\sum }}U_{i}T_{i}$$

where *D*_3_ represents the demand for industrial grains and *U_i_* refers to the grain consumption of the five main related industries in wine brewing, alcohol production, starch processing, soybean crushing, and non-staple food processing. *T_i_* indicates the grain consumption coefficient per unit of industrial product *i.* Industrial grain is also a form of indirect use, so we use the conversion rate to calculate it.

#### 2.1.4. Seed Use

Seeds are used as raw material for the following year’s sowing, whose demand is mainly determined by scales of sown areas and advances in storage or breeding technology, and accounted for less than 5% of the total. It was calculated as:

(5) $$D_{4}=\overset{15}{\underset{i=1}{\sum }}S_{i}G_{i}$$

where *D*_4_ represents the grain consumption for seeds, *S_i_* is the sowing volume per unit area of the fifteen kinds of grains, and *G_i_* is the sown area. Grains for seed use is a way of indirect use that can be calculated using the conversion rate.

#### 2.1.5. Losses

Grain losses are the amount lost during the production, storage, circulation and consumption of grains and were counted as follows:

(6) $$D_{5}=\overset{15}{\underset{i=1}{\sum }}L_{i}$$

where *D*_5_ represents the total amount of grain losses and *L_i_* indicates the loss of cereals, soybeans, potatoes, etc.

The above five different uses together constitute China’s total grain demand.

#### 2.1.6. Per Capita Grain Demand

To simulate the grain demand at the demographic peak in China, we considered two major factors (using factor analysis): per capita grain demand and population size. In particular, per capita grain demand is determined by income, grain prices and individual characteristic factors, e.g., gender, ethnicity, and dietary preference, which can be calculated as:

(7) $$Y_{t}=\alpha I_{t}+\beta R_{t}+\gamma O_{t}+\varepsilon_{t}$$

where *Y_t_* represents per capita grain demand in year *t*, *I_t_* stands for per capita income, *R_t_* for the grain prices, *O_t_* for individual characteristics, and *ε* for random error.

### 2.2. Grain Supply Model in China

The improvement in the grain supply capacity is essential to satisfy grain demand. The grain supply model was thus proposed based on grain production, imports, exports, and stores in accordance with the FAO classification standards [39]. The model incorporated non-linear time trends, which comprised a combination of linear terms, all modeled hierarchically. The first level was the calculation of grain production, to which we progressively added the grain trade acting as a critical factor affecting total supply at the next level. However, the unique situation in China was a massive grain store, an influencing factor with a significant elasticity factor for grain supply. Employing the selected data, the grain supply model was calculated as follows:

(8) $$GSQ_{i}^{t}=GPQ_{i}^{t}+I_{i}^{t}-E_{i}^{t}+VS_{i}^{t}$$

where $GSQ_{i}^{t}$ is the grain supply of *i* crops in year t and $GPQ_{i}^{t}$ is the grain production of *i* crops in year t. $I_{i}^{\left(t\right)}$ and $E_{i}^{\left(t\right)}$ represent the volume of grain imports and exports, respectively, and $VS_{i}^{\left(t\right)}$ represents the change in grain store reserves.

As a key component of the grain supply, grain production volume means the actual amount of grain output in one year and refers to a function of the grain yield level (per unit area), cultivated area, and multi-cropping index. Here, grain productivity relates to the proportion of irrigated farmland, the use rate of fertilizers, agricultural films and improved varieties as a production function, expressed as:

(9) $$Y_{1}=\beta_{0}X^{\beta_{1}}X^{\beta_{2}}X^{\beta_{3}}\ldots X^{\beta_{k}}$$

(10) $$GPQ_{i}^{t}=P_{i}Y_{i}$$

where $Y_{i}^{t}\;$ is grain yield, *X* is factors affecting *Y*, $GPQ_{i}^{t}$ is the production of *i* crops and $P_{i}^{t}$ denotes grain acreage.

### 2.3. The Analysis Framework of Grain Supply and Demand Scenarios

This paper proposes an analytical framework based on supply and demand balance. On the demand side, 3 population sizes and 3 per capita demand levels are combined into 9 demand scenarios.

The per capita demand was calculated by three methods: (1) the low scenario is based on the dietary balance theory and estimates the per capita demand of meeting the minimum nutritional standards; (2) the medium scenario is based on extrapolating the historical grain demand changes in China over the past 58 years; and (3) the high scenario is based on the reference per capital grain demand of some Asian developed countries or regions, i.e., Japan, Korea, Taiwan of China, and Hong Kong of China, which have similar dietary structures as mainland China, and intercepted the same period of gross domestic product (GDP) change (China’s GDP is expected to increase from USD 8772 to USD 37,877 in 2020–2050).

**Figure 2:**
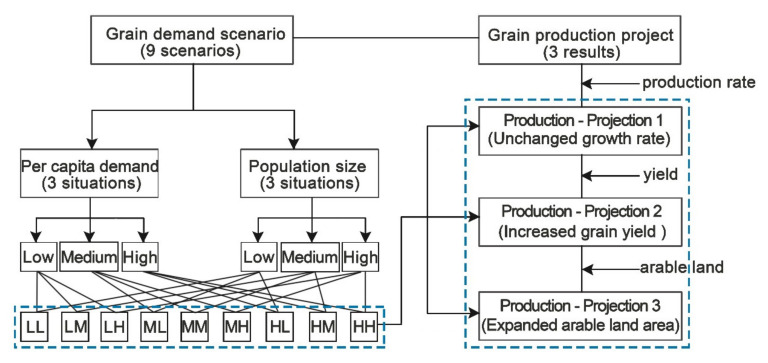
The analysis framework of grain demand scenarios and production projection.

Considering three different per capita grain demand situations (high, medium, and low) and three varying population size situations (low, medium, and high), we build a 3-by-3 scenario matrix (Figure 2): to begin with, we build the demand scenarios 1-LL, 2-LM, and 3-LH projected based on different per capita grain demands under low population size situations; next, by altering the population size to the medium level, we predict demand scenarios 4, 5, and 6 corresponding to low (ML), medium (MM), and high (MH) per capita situations, respectively; finally, scenarios 7, 8, and 9 present various demand possibilities of per capita grain demand under the high population scenario.

In the grain supply side, we set up three prediction plans based on time series analysis: (1) Projection 1 assumes that China’s grain production maintains constant growth rate; (2) projection 2 is considering the yield increase potential based on Projection 1; (3) Projection 3 is further added to the arable land area expansion potential on the basis of Projection 2.

In the demand–supply balance section, we focused on the following issues: (1) which demand scenarios will be met if the current growth rate of grain output in China remains unchanged (Projection 1)? (2) Which demand scenarios will be further satisfied if grain yields are set to be enhanced (Projection 2)? (3) Will all the demand scenarios be fulfilled if the cultivated area is further enlarged from P2? If not, which scenarios will be considered risky for China’s grain self-sufficiency and even global food security? Overall, a bidirectional multi-scenario projection model is employed to analyze the gap between grain production and demand during the peak population period in China.

### 2.4. Data Source

We employed grain production, trade, and store data from Food and Agriculture Organization (FAO) databases and the China Statistical Yearbook, dated up to 2018, which describe the availability of different categories of grains for human consumption [39,40,41]. We used annual population, cultivated area, crop yields, and harvested area for the years 1961–2018 from the China Statistical Yearbook and proofread all data combining the World Bank databases. We employed all data from the provided data from 1961–2018 to analyze the Chinese grain system from the viewpoint of production and demand and predict future trends and global implications [42,43]. In particular, two sectors of data are needed to quantify the relationship between supply and demand as follows:

(1) China population projection data from World Population Prospects 2019 were drawn to simulate three different population scenarios [37].
(2) Recent studies have shown that more grain losses in consumption were objectively present in China, and the data were mainly from the FAO food balance sheets [44,45]. We will not discuss the impact of changes in the databases; for example, the data balance sheet was updated in 2014, so there are two different sets of data from 1961–2013 and 2014–2018.

What needs to be pointed out is that the definition of grain in China differs from the general international understanding. The FAO defines grains to include mainly wheat, maize, rice, and other cereals, while China also included potatoes and soybeans in its calculation [39,40,41]. This is because China is the world’s largest producer of potatoes and has recently implemented a policy to promote potatoes as a national staple food, and meal from soybeans is the main feed source for the Chinese breeding industry. Hence, according to the Chinese Statistical Yearbook, potato and soybean are converted into unprocessed grains at ratios of 5:1 and 1:1, respectively. With this, we analyzed the demand and production components of the grain in simulations.

## 3. Results

### 3.1. China’s Grain Demand during 2020–2050 under Different Scenarios

#### 3.1.1. Per Capita Grain Demand for the Period 2020–2050

Considering a range of assumptions about China’s per capita grain demands, we modeled three scenarios: high, medium, and low. In the low scenario, we assumed that China would only need to meet the basic nutritional requirements in 2050. According to the standards published by the Chinese Nutrition Society, this implies a daily per capita intake of 250–400 g of cereals and potatoes, 40–75 g of livestock and poultry meat, 40–75 g of aquatic foods, 40–50 g of eggs, 300 g of milk, 25–35 g of soybeans, and 25–30 g of edible oil, corresponding to revised grain consumption coefficients of 1, 3, 1, 1.8, 0.5, 1, and 3, respectively. Calculatedly, this scenario’s per capita grain demand is 389 kg, which does not account for grain losses and possible additional consumption due to individual diets and habits. The results estimated by this method are slightly lower than those of existing studies [34], but it can be regarded as the bottom line of per capita food demand in China (Table 1).

In the medium case, referring to historical trends and existing studies, we counted five categories of grain consumption and estimated China’s per capita grain demand at 517 kg. In particular, the per capita demand for food is predicted to be 170 kg, and its decreasing trend is expected throughout the projection period. Grains for feed reach 236 kg. Industrial grains represent 87 kg based on the consumption of the five major processing industries. Seeds and per capita grain losses account for 12 kg, with the latter showing a more distinct decline from the current amount (19 kg). We attribute this to improving the grain management system and implementing the Anti-Food Waste Law in China. The high scenario was designed taking into account the growth of developed East Asian economies, i.e., Japan and South Korea, featuring similar dietary structures.

We hypothesized that GDP primarily influences the increase in per capita grain demand. Combining the World Bank and scholars’ projections of GDP growth rates for 2020–2050 [41], we speculated that China’s GDP per capita would grow from USD 8772 to USD 37,877. For the same amount of GDP growth, Japan and South Korea raised their per capita food demand by 2% and 3.8%, respectively. For the past 60 years (1961–2020), China’s per capita grain demand has grown at an annual rate of 1.6%, compared with 0.4% in Japan and 0.5% in South Korea. As a result, we predicted that China’s per capita grain demand in 2050 in the high scenario would increase by 10% from 2020 to 546 kg. Using this approach, we mainly consider the influence of economic factors and dietary habits on per capita grain demand [35]. As the calculation was bound to the previous production technology of the reference subjects, we possibly overestimated the amount of grain for feed and processing. Nonetheless, it will not affect its role as an upper limit value for China’s per capita grain demand in our design scenarios.

#### 3.1.2. Population Size Projections from 2020 to 2050

**Figure 3:**
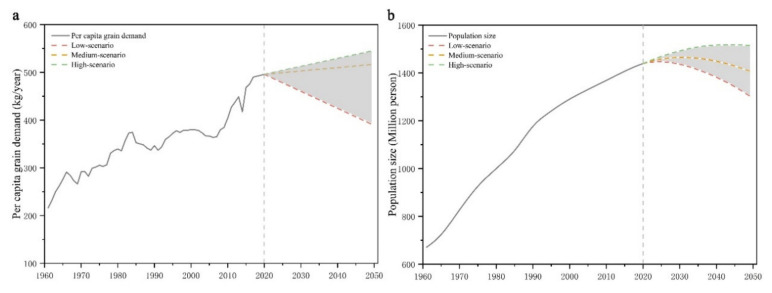
Scenarios of per capita grain demand (**a**) and population size (**b**) during 2020–2050.

We projected demographic data based on the World Population Prospects database, which is divided into three main scenarios: high, medium, and low scenarios (World Population Prospects 2019). The estimates are based on available sources for individual countries on population size and fertility, mortality, and migration levels. In the high scenario, China’s total population grows at an annual rate of 0.2% from 2020 to 2044, peaking at 1.52 billion in 2044 and declining gradually to a total population of 1.5 billion in 2050. In the medium scenario, China’s population peaks in 2031 at 1.46 billion, with the same projected trend of first increasing and then decreasing, reaching a minimum value of 1.4 billion in 2050. In the low scenario, China’s population will continue to decline after 2024, with a total population of merely 1.29 billion by 2050 (Figure 3). The population size and trends in the three scenarios are significantly different. In particular, the 200 million population difference between the high and low scenarios at the end of our study period is expected to profoundly impact the projection of total grain demand.

#### 3.1.3. China’s Grain Demand during 2020–2050 under Nine Scenarios

**Figure 4:**
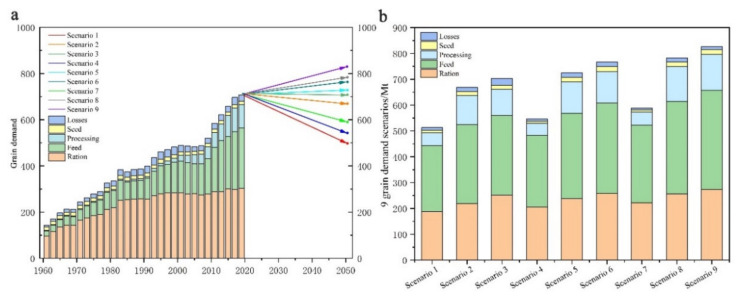
Scenario simulation of 9-grain demand (**a**) during 2020–2050 and comparison of 9 scenarios in 2050 (**b**).

The simulation results of grain demand show that the gap between the total demand of the nine scenarios in 2050 reaches 323.8 Mt. If we calculate the per capita demand of 517 kg according to the medium scenario, this gap is equivalent to the annual food consumption of 620 million Chinese people (Figure 4a). The estimated total grain demand in 2050 under scenario 1-LL would decline by 22.8% due to the dual coupling of low population and low per capita volume. However, scenario 9-HH suggests that a 15.8% increase in grain production (ca. 113 Mt) would be needed to meet the 2050 scenario. If demand reflected these two extremes, both the quantity and structure of China’s grain production would suffer considerably.

In Scenario 1-LL, China’s total grain demand in 2050 is calculated to be only 503 Mt, comprising 188 Mt for food (37.4%), 255 Mt for feed (50.7%), and 60 Mt for processing and seeds. Such a demand structure is similar to that of countries such as Europe and the US, featuring a lower total grain demand but a higher share of feed grains, and more significant consumption of meat is expected to stimulate the growth of feed grain consumption. Although per capita meat consumption in China has increased by 50% over the last 24 years, the Chinese still have a predominantly plant-based diet and are unlikely to reach the levels of Europe and the US individually. Hence, this scenario is applicable only if the food consumption structure in China is Westernized and the population size remains at a low level. In Scenario 2-LM, the set change in population size is the same as in Scenario 1, but the per capita grain demand (projected at the end of the study period) is 128 kg higher, or 21 kg above the current figure. Even so, the projection of total grain demand in 2050 falls by 45 Mt and reaches 669 Mt due to the projected decline in population. Here, food grains, feed grains, and industrial grains account for 32.8%, 45.6%, and 16.8%, respectively, comparable to China’s current grain consumption structure. Specifically, food is expected to decline by 40 Mt, but feed grains are expected to increase by 53 Mt, which is compatible with the trend that meat consumption will further grow to drive a continuous increase in feed grains. In Scenario 3-LH, the total demand is 706 Mt, which is numerically comparable to the current amount. However, there are set changes in both factors, per capita demand and population size. In particular, the population size is projected to change as in the previous scenarios, while the per capita food demand increases by 50 kg compared with the current level. This increase is mainly attributed to 12% for feed and 11.7% for industrial grain, with little change in food grains. We attribute this scenario of high per capita demand and low population size to the effects of growing consumption of meat, eggs, milk and processed foods due to urbanization, on the one hand, and rising mortality due to an aging population on the other.

In Scenario 4-ML, China’s total grain demand in 2050 is 546 Mt, when the total population is projected to change modestly. The shift in per capita grain demand in this scenario is similar to Scenarios 1-LL, decreasing from 496 kg to 389 kg, suggesting a Westernization of the grain consumption structure. Specifically, there is no significant variation in structural characteristics, with total food amounting to 205 Mt and feed grains to 278 Mt. Catering to this situation requires a more intensive grain consumption pattern and a series of grain policies in line with nutritional norms. In Scenario 5-MM, assuming a mid-sized demand per capita and population, the total grain demand in 2050 reaches 725 Mt, up 11 Mt compared with 2020. At the end of the projection period, the total food use decreased by 21.1% to 238 Mt, feed grains increased by 25.9% to 330 Mt, and industrial food use increased by 18.7% to 122 Mt. This scenario is comparable to the current trend in China, with total grain demand reaching a peak of 740 Mt in 2036. Such an “inverted U-shaped” variation also appears to mirror the findings of prior studies [33,34,35]. In Scenario 6-MH, the simulated total grain demand increases to 766 Mt, and the increase is due to a 29 kg uplift in per capita demand. Although the structural change in grain demand is not significant, the relatively rapid growth in per capita demand is not consistent with the current reality of a gradual decline in ration consumption. However, it is possible to see an increase of 51 Mt in total demand, as the indirect consumption of grain is related not only to changes in total volume but also to processing technology and farming practices.

Scenario 7-HL projected a total food demand of 589 Mt in 2050, which is the joint result of a high population growth pattern and low per capita grain demand. Admittedly, with the current population growth rate of 0.4–0.5%, it will be difficult for China’s population to grow above 1.5 billion in 2050. However, the demographic growth potential released by China’s gradual easing of fertility restrictions cannot be ignored either. In this scenario, feed grain demand increases to 300 Mt. Apart from the total population increase, this is also indirectly influenced by the growth in per capita meat consumption in the low scenario. In Scenario 8-HM, the per capita food demand shifts to the medium scenario, and the total amount is predicted to increase by 194 Mt to 783 Mt. This value needs to be reached at an annual growth rate of 0.5%, which is lower than the actual growth rate of China at present. However, it is still explainable when referring to Japan, Korea, and the United States. The growth rate is characterized by a rapid increase followed by saturation and then a gradual decrease. In addition, the forecasts of food and feed grains are 257 Mt and 357 Mt, respectively, where the increase in feed grains is related to the growth in total population and per capita demand. In Scenario 9-HH, total demand is the maximum of the scenarios at 827 Mt due to the combined effect of high per capita demand and a high population growth pattern. The total food demand is projected to increase by 15.8%, mainly in feed grains and industrial grains rather than in food use. In this scenario, it is difficult for China to reach the 95% self-sufficiency rate in grains proposed by the Chinese government, which would require a reduction in the nonessential demand share, including losses throughout the process.

### 3.2. China’s Grain Production under Different Projections

#### 3.2.1. Change Trend of Yield and Arable Land Area of China’s Grain Production

The gradual improvement in grain production conditions is the basis for supporting the rapid growth in grain demand. The growth rates of grain yields of the three major categories of grain crops—cereals, potatoes and soybeans—from 1961 to 2018 were all above 90%, especially wheat, which increased 8.7 times, while yams (converted into sweet potatoes) changed relatively slowly, increasing by only 91%. Overall, rice, maize and wheat had the highest and fastest-growing yields of the three major staples, which have become the main drivers of China’s soaring grain production. In contrast to the rapid growth in grain yields, the area under grain cultivation changed little, increasing by only 0.61 Mha over 58 years. From 1961 to 2014, the area under grain cultivation declined by 5% and merely rebounded with the implementation of land preservation policies such as high-standard farmland in 2013.

The significant feature of the change in grain planting area is that the proportion of the three major staple foods has been increasing year by year and accounted for 83% by 2018, which is an important support for the growth of grain production. The decrease in the area planted with potatoes and beans also shows that China has always adhered to the food security policy of “ensuring basic self-sufficiency of grain and absolute security of staple food”. During the study period, China’s total grain production increased 3.7 times, with the largest increase of 13.3 times for maize, mainly because the increase in demand for maize as an industrial food stimulated the expansion of production. The main reason is that the increase in demand for maize as an industrial grain stimulated the expansion at the production end. Wheat and maize increased 8.2 and 3 times, respectively, to secure the supply of food. Despite a 65% increase in potato production, the share of total grain decreased from 12.5% to 4.4%, a result of the restructuring of other grain crops due to increased production of China’s three major staples. While the demand for edible soybean oil and soybean meal (for feed) has increased, soybean production has risen at a lower rate, and the supply–demand gap has relied mainly on the international market to resolve.

Overall, over the past 60 years, China’s grain production capacity has increased significantly while also gradually strengthening the main position of the three staple grains, which is the combined result of current Chinese production conditions and grain policies.

#### 3.2.2. China’s Grain Production Simulation under Different Projections

**Figure 5:**
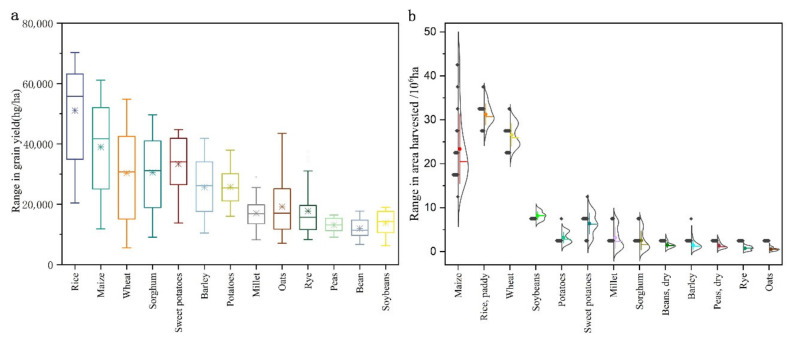
Ranges in grain production yield (**a**) and arable land area (**b**) during 1961–2018.

This section simulates three production alternatives based on different factors affecting grain productivity. China’s grain yield level has increased rapidly over six decades (Figure 5a). In the case of developed countries, although the increase in grain yields per unit is expected to slow down gradually, the overall upward trend will be maintained; therefore, the grain yield increase is taken as an influencing factor. China is currently losing its high-quality arable land resources rapidly, but the implementation of active land preservation policies in China is expected to improve this situation. Therefore, we consider improving arable land resources as the second factor affecting productivity enhancement and present the following projections.

**Figure 6:**
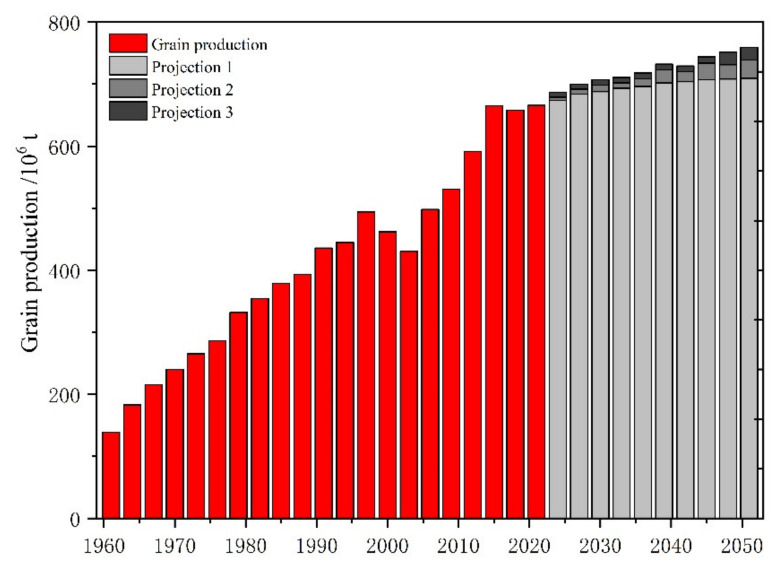
Projection of 3-grain production during 2020–2050.

Projection 1: Here, we project that increases in grain production maintain their current pace (P1). These increases will be the combined result of various factors, mainly the adjustment of the cropping structure. Over the past 58 years, grain output in China has increased at an average rate of 1.4% per year, but the growth rate has declined to 0.62% in the last decade and has decreased at a rate of approximately 0.5% per decade. Projection 1 assumes that grain production growth rates will decrease by 0.1% per decade to reach 709 Mt in 2050, an increase of 51.2 Mt compared with the current value (Figure 6). As the base option for our proposal, we set a relatively small increment.

Projection 2: This alternative enhances the setting of the grain yield variable (P2). Assuming that China’s grain yields continue to grow at the current rate, calculated as between 1–2% based on the 10-year average, total grain production in 2050 will increase by 29 Mt to 738.6 Mt from P1. Projection 2 hypothesizes that the trigger for the simulation will be an increase in yield efficiency due to innovations in agricultural technologies.

Projection 3: Based on P2, we further consider the improvement of arable land resources. The amount of China’s grain arable land has increased at an annual rate of 1% since 2013, while China’s existing policy plans to build 1.2 billion mu of well-facilitated farmland by 2030 to guarantee grain production capacity. In Projection 3, China’s grain production is predicted to increase by 20.4 Mt from P2 to 759 Mt, which are predicted to be the combined result of higher yields and arable land policy support (Figure 6).

### 3.3. China’s Grain Demand and Production Balance during the Population Peak

**Figure 7:**
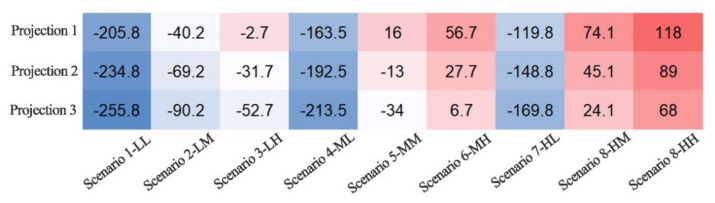
The gaps of demand scenarios and production projection in 2050. If demand is greater than production, the gap is positive and denoted in red. Conversely, if demand is less than production, the gap is negative, indicated in blue.

A basic balance between grain supply and demand is a strong guarantee for China’s food security and an important prerequisite for the sustainable development of the global food system. Although China has maintained an overall balance over the past 58 years, it is bound to break down in the future with the growth of total grain demands triggered by the dietary structural transition. The grain demand scenarios show that the total demand varies up to 323.8 Mt, which is estimated based on multiple related factors, providing more possibilities to accurately analyze the trends in the next 30 years. The three projections of grain productivity are a comprehensive analysis in which we gradually add controllable variables. Based on the matches of different scenarios and projections, we will discuss how to address the gap between grain demand and production in three comparative ways (Figure 7).

Simulation 1: With the current setting of grain output increasing at an average of 1.4% per year, Scenario 1 projects that China will produce 709 Mt of grain in 2050, which will meet the grain demand of Scenarios 1-LL, 2-LM, 4-ML, and 7-HL. The difference between the projected grain production of Projection 1 and the demand of Scenario 5-MM is only 16 Mt, while it differs from the demand of Scenario 9-HH by 117 Mt (Table 2). The gap with Scenario 9 is one-fifth of China’s total grain production and the annual grain consumption of 230 million people. If it maintains the current level of grain yields, additional arable land of approximately 25 Mha will be required to address the gap, further exacerbating the shortage of arable land resources and posing a significant challenge to agricultural production in China. Assuming that China’s net grain imports remain at the current level of approximately 90 Mt, over 80% of the additional demand will be filled. Only 4 Mha of additional arable land will be needed, suggesting that sufficient and stable grain imports are necessary to guarantee the production–demand balance. If net grain imports were to remain at only 75% of current levels in 2050 (equal to a scenario where import source countries exclude the US, Australia, and Canada), it would likely result in a 43 Mt grain shortage and additional requirements of approximately 9 Mha of arable land resources in domestic China. If the regulatory role of grain stocks is fully utilized, the grain supply and demand gap may be filled, and the pressure on China’s land resources can also be eased.

Simulation 2: Innovations in agricultural technologies contribute to the growth of grain yields. Assuming that the growth rate of grain yields will remain between 1–2% in 2030–2050, the grain production simulated in Projection 2 will reach 738 Mt in 2050, meeting the grain demand of Scenario 5-MM further than that of Projection 1 (Table 2). In particular, the shortage from Scenario 6-MH is merely 27 Mt and can probably be solved by domestic production or grain imports. The difference from Scenario 8-HM is approximately 45 Mt, equivalent to the annual grain demand of 87 million people. Nevertheless, the capability of net grain imports is expected to fill the shortage, and the balance of grain supply and demand can still be sustained to ensure China’s food security. However, there is still a supply–demand gap of 89 Mt between Projection 2 and Scenario 9-HH. To achieve the 95% grain self-sufficiency level proposed by the Chinese government, even if yields were to increase by 1–2%, an additional 17.4 Mha of arable land resources would still be required for grain production. The additional needs are equivalent to 15% of the current total cultivated land areas. Although not all of the predicted grain demands are met by increasing yields, the raised grain production can feed an extra 56 million Chinese people. Therefore, enhancing grain yields has become one of the key measures for the Chinese government to reduce the gap between grain production and demand.

Simulation 3: Arable land is the carrier of grain production, and its quantity determines the area sown for grains. Projection 3, based on Projection 2, further assumes that the total amount of arable land for grain production increases at a rate of 1% per year, reaching a total of 759 Mt in 2050 (Table 2). Specifically, the difference between this projected volume and the grain demand from scenario 6-MH is only 6 Mt, accounting for approximately 6% of China’s current total grain imports, equivalent to the output of 0.2 Mha of arable land, less than 0.2% in China. Compared with demand scenario 8-HM, a shortfall of 24 Mt of grain production is projected, representing merely a quarter of current grain imports, which can easily be filled. When the projected demand for grain reaches a maximum (Scenario 9-HH), there will be a 68 Mt production shortage for Projection 3, approximately one-tenth of China’s average annual grain demand. With a 95% self-sufficiency rate as the target, an additional 14.5 Mha of arable land is needed to achieve a balance between supply and demand. This will put enormous pressure on China’s agricultural production. By then, grain imports are also expected to serve as an essential means of supplementing China’s grain shortage. In general, when grain productivity reaches the Projection 3 assumption, China’s grain demand scenarios will be basically satisfied, and the supply–demand balance will be effectively sustained through domestic production, grain trade, and stock regulation.

## 4. Discussion

Addressing China’s grain supply and demand over the next 30 years while ensuring the stability of the global food system is one of the key challenges facing global food sustainability [49]. China’s demand for grains for human consumption and livestock feed is likely to continue to increase due to increased consumption for animal production [50,51]. The results of this study reveal that feed grain is about to overtake food grain as the largest use of grain, due to the change in diet that has come with dramatic economic reforms [52]. Therefore, following a healthy diet with moderate consumption of animal production would also reduce the pressure on cereal supply [53]. In addition, a more efficient grain supply chain is also necessary, including improving the output efficiency of water and arable land resources. The grain stocks and trade have also played an important role for China’s grain supply, but the stability of the international food trade network has been greatly impacted by trade frictions and COVID-19 [54,55]. The restrictive trade system has compromised the continuity of supply and access to food [18]. Therefore, it is important for China to fulfill the grain supply by domestic production in future.

Over the past six decades, China’s grain yields have increased by an average of 350%, which is the key to the improvement of grain self-sufficiency. It is also the result of a combination of agricultural production technology, the use of farm machinery, and an increase in agricultural input and policy incentives. Even so, there is a certain gap be-tween China’s grain yield levels and those of developed countries, which is partly at-tributed to the lower efficiency of China’s smallholder farming system. With full consideration of topography, climate and other natural factors, China’s grain yield level will have the possibility of further improvement, which is one of the future keys to solving the gap between grain supply and demand.

To explore China’s grain production potential, China should strengthen the financial inputs and subsidies for grain production and establish a comprehensive assessment of its production, distribution, surplus, shortage transfer and management system. It is clear from the simulation results of grain production that increasing scientific inputs in agriculture and innovating production technologies are also not negligible. These are essential building blocks for improving grain yields. In addition, the food production process also needs to address the environmental burden caused by the overuse of chemical fertilizers, pesticides, and mulch [56,57]. Last but not least, these results also illustrate two significant problems in the current food production–consumption process: on the one hand, structural reforms on the supply side should be carried out to ensure appropriate stock levels; on the other hand, the amount of grain waste reaches 18 Mt annually, which requires economic and scientific use of grain to reduce losses and waste [58].

In the context of rapid urbanization, China is losing its well-facilitated arable land resources. In response, China practices the strictest farmland protection system and a strategy of sustainable farmland use to increase farmland productivity. In light of this, the government has also implemented a well-facilitated farmland construction policy since 2013, and in 2020, the enhanced 800 million mu of well-facilitated farmland has allowed China to raise its grain productivity by 10–20%. Although the interventions have to some extent protected arable land resources and improved its utilization rate, China’s arable land is still under threat of soil erosion, sanding, salinization, and environmental pollution. In turn, these problems have become major obstacles to the further enhancement of arable land. They also set up greater difficulties for the realization of Projection 3.

With continuing economic growth and increasing aggregate demand for grain, related studies also illustrate that supply-side reforms in grain are significant in developing countries [59,60,61]. The paper raises additional possibilities for modeling sustainable grain demand scenarios and production scenarios to ensure food security. It is an analytical framework that can be applied to other countries with large populations. In addition, a multi-scenario simulation of grain demand and production balance is expected to enable more accurate control of the Chinese grain system during the population peak. Solutions to China’s grain supply and demand problems will help shift the global food security problem to a healthier and more environmentally sustainable development.

## 5. Conclusions

Combining various econometric approaches, we proposed a basic framework for simulating China’s food supply and demand in 2020–2050. Within this framework, we systematically modeled nine grain demand scenarios and three grain production projections by considering various relative factors such as dietary structure changes, population size, yield improvement, arable land resources, and grain imports. It can provide some references for developing forward-looking measures to ensure future food security in China and the world.

China’s grain demand will be between 503 and 827 Mt from 2020 to 2050. This study simulated nine scenarios of China’s future grain demand (2020–2050) based on historical population size and per capita grain demand data (provided by FAO from 1961 to 2018) in this study [62,63,64]. The results show a difference of 323.8 Mt between scenario 9 (the highest demand) and scenario 1 (the lowest demand) and 221.8 Mt higher than scenario 5, the most stable demand. These large differences are the great openness of our simulation projects, where we consider multiple scenarios and discuss the possibilities. In scenario 9-HH, China’s total grain demand will increase by 113 Mt (15.8%) compared with 2020, and this scenario is the result of a combination of rapid population growth and dietary transition. However, among the other six scenarios (except scenarios 1-LL, 9-HH, and 5-MM), scenario 8-HM had the largest increase in total volume at 9.7%, and scenario 4-ML had a decrease rate of 23.6%.

Among the nine scenarios, Scenario 1-LL, Scenario 4-ML, and Scenario 7-HL, which have low total demand, are formed by the population size and low per capita grain demand. These requirements are calculated in full compliance with nutritional standards and do not consider the actual excess consumption. Therefore, all three scenarios serve as the bottom line of demand, albeit with probable occurrence. Scenario 2-LM, Scenario 3-LH, and Scenario 3-MM show moderate predictions, more in line with the current trend of China’s grain demand. Scenario 6-MH, Scenario 8-HM, and Scenario 9-HH are assumptions based on China’s high population and average demand. All three scenarios, although less likely, are still possible with China’s proactive population policy in place and its rapid growth in indirect per capita grain demand.

On balance, the relatively stable changes are more appropriate for the historical trends and current situation of the nine grain demand scenarios in China. China’s grain production will be between 709 and 759 Mt during 2020–2050 under the three projections of this study. The results also demonstrate that the three projects of grain production show a large variation when different influencing factors are added. When grain production maintains only the current growth trend, project 1 simulates grain production of 709 Mt in 2050. When further considering a 1–2% increase in grain yields, project 2 predicts a grain production of 738 Mt. If changes in arable land are included, the grain production would further increase to 759 Mt.

This study also shows that there was still a gap between the three scenarios of grain production and the nine scenarios of demand in China, which is becoming a research focus that needs attention at present [65]. Comparing the nine scenarios with project 1, the grain production capacity in 2050 will meet the demand of scenarios 1-LL, 2-LM, 4-ML, and 7-HL, but there is a difference of 117 Mt with scenario 9-HH and 49 Mt with the other three scenario averages. In project 2, the improved agricultural production technology contributes to an increase in total grain production by 29 Mt. The total grain production differs from Scenario 9-HH by 89 Mt in 2050, indicating that it is difficult to supplement the production–demand gap by increased grain production alone. However, this additional grain production will feed more than 40 million Chinese people and is one of the measures to alleviate the contradiction between production and demand for the Chinese government. In project 3, the grain production in 2050 will increase by another 20 Mt, which already exceeds the grain demand of Scenario 6-ML and differs from that of Scenario 8-HM by only 24 Mt. However, although a fundamental production–demand balance was reached, the scenario was the upper limit of food production in China. This study has proven that even if arable land and yields reach a certain level in 2050, there is still a certain gap with the relatively stable demand scenarios 8-HM and 9-HH. This will consume much more arable land resources to support grain production, which will put tremendous pressure on China’s agricultural system. In addition, our modeling of production does not consider the reductions due to natural disasters such as floods, droughts, and climate change, which will further aggravate the instability of food production and expand the food gap [66,67,68].

The study also has some shortages that need to improve in further studies. While stimulating China’s overall grain demand and production scenarios, we did not consider intra-China variations in this paper. For example, population age structure determines grain demand changes or urban–rural population disparities that trigger direct and indirect demand differences. These would also be a direction for future refinements in research. In addition, grain supply is subject to multiple uncertainties, with natural disasters directly affecting production and national conflicts limiting grain trade. Due to the lack of sufficient information, the above uncertainties are also not considered by this paper. Considering the complexity of the food security problem, we did not look for a single solution but compared different alternatives under various possible scenarios.

## Figures and Tables

**Table 1:** Comparison with the results of existing studies.

Scenario	Method	References	Result	Difference
Low	Dietary balance estimation	Tang et al. [34]	386	−3
		Xin et al. [30]	386	−3
Medium	Historical trend extrapolation	Feng et al. [46]	450	−67
		Lin et al. [47]	470	−47
High	International Experience Study	Xin et al. [48]	517	0
		Huang et al. [33]	531	14

**Table 2:** 9 Scenario simulation values for grain demand and production in 2050.

	Indicator	Scenario Analysis								
		Scenario1 LL	Scenario2 LM	Scenario3 LH	Scenario4 ML	Scenario5 MM	Scenario6 MH	Scenario7 HL	Scenario8 HM	Scenario9 HH
Demand	Population size (billion people)	1.29	1.29	1.29	1.4	1.4	1.4	1.51	1.51	1.51
	Grain per capita demand (kg/capita/year)	389	517	546	389	517	546	389	517	546
	Total grain demand (million tons)	503.2	668.8	706.3	545.5	725.0	765.7	589.2	783.1	827.0
Production	Projection 1	709.6	709.6	709.6	709.6	709.6	709.6	709.6	709.6	709.6
	Projection 2	738.7	738.7	738.7	738.7	738.7	738.7	738.7	738.7	738.7
	Projection 3	759.0	759.0	759.0	759.0	759.0	759.0	759.0	759.0	759.0
Balance	The gap with current production (Mt)	154.8	−10.8	−48.3	112.5	−67.0	−107.7	68.8	−125.1	−169.0
	The gap with production projection 1 (Mt)	205.8	40.2	2.7	163.5	−16.0	−56.7	119.8	−74.1	−118.0
	The gap with production projection 2 (Mt)	234.8	69.2	31.7	192.5	13.0	−27.7	148.8	−45.1	−89.0
	The gap with production projection 3 (Mt)	255.8	90.2	52.7	213.5	34.0	−6.7	169.8	−24.1	−68.0
	The average gap (Mt)	232.1	66.5	29.0	189.8	10.3	−30.4	146.1	−47.8	−91.7
	The gap to fill by grain imports	-	-	-			30%		50%	100%

## Data Availability

This study did not report any data.
